# Conjugation of Pea Peptides and D-Xylose via Maillard Glycosylation and Its Functionality to Antagonize Alcohol-Induced Liver Injury in Zebrafish

**DOI:** 10.3390/nu17152570

**Published:** 2025-08-07

**Authors:** Guanlong Li, Xiaolan Liu, Siyu Diao, Xiqun Zheng

**Affiliations:** 1 Heilongjiang Provincial Key Laboratory of Corn Deep Processing Theory and Technology, College of Food and Bioengineering, Qiqihar University, Qiqihar 161006, China; 03580@qqhru.edu.cn (G.L.); 18545049329@163.com (S.D.); 2 College of Food Science, Heilongjiang Bayi Agricultural University, Daqing 163319, China

**Keywords:** pea peptides, Maillard glycosylation, pea glycopeptides, antagonize alcohol-induced liver injury

## Abstract

Background: In this study, the preparation of pea glycopeptides based on the Maillard glycosylation pathway (PPH-M) and its antagonistic mechanism against alcoholic liver injury in zebrafish were studied. Results: The results showed that the conjugation of D-xylose significantly improved the antioxidant activity of pea protein hydrolysates (PPHs). The structural characterization indicated that PPH was successfully covalent binding to D-xylose, which was mainly manifested as a stretching vibration change in Fourier transform infrared spectroscopy (FTIR) and molecular size increase. Scanning electron microscopy (SEM) and zeta potential also confirmed the covalently bound of the two. In addition, a model of alcohol-induced liver injury in zebrafish was established. Through the intervention of different doses of PPH-M, it was found that the intervention of PPH-M could significantly increase superoxide dismutase (SOD) activity, reduce malondialdehyde (MDA) content, aspartate aminotransferase (AST), and alanine aminotransferase (ALT) activity, and significantly improve alcohol-induced liver injury in zebrafish. The protective effect of PPH-M was also confirmed by liver pathology and fluorescence microscopy. Finally, reverse transcription-polymerase chain reaction (qRT-PCR) results indicated that PPH-M could significantly regulate the expression level of antioxidant-related mRNA. PPH-M could also regulate the expression of the Keap1/Nrf2 signaling pathway and up-regulated glutathione synthesis signaling pathway to antagonize alcohol-induced liver injury in zebrafish. Conclusion: This study revealed the mechanism of PPH-M antagonized alcoholic liver injury and laid a theoretical foundation for its development as functional foods.

## 1. Introduction

Pea is the second largest edible bean, with an annual output of about 13.5 million tons in the world. China is the second largest producer of peas in the world, with an annual output of about 1.5 million tons [1]. Compared with other legumes and grains, peas have a high protein content of about 20%, including 55% globulin, 20% albumin, and about 10% gluten [2]. Pea protein has high nutritional value and is rich in eight essential amino acids for the human body, and its amino acid composition is close to the FAO/WHO recommended pattern [3]. Therefore, the development and application of pea protein have attracted much attention in the food industry. Due to the poor solubility and digestibility of pea protein, the utilization rate of pea protein is low. Studies have found that the active peptide of pea protein prepared by biological methods not only improves its solubility, digestion, absorption, and utilization but also endows it with unique physiological activities, such as antioxidation, anti-hypertension, immune regulation, and anti-inflammation [4,5,6,7]. Although existing studies have shown that pea protein peptides have good antioxidant activity, there is still a gap compared with commercial antioxidants. The antioxidant activity of the active peptide can be significantly improved by modification of the active peptide by biotechnology.

Glycosylation modification is the covalent linkage of a carbohydrate containing a hydrophilic group to a protein or peptide molecule. The glycosylated products have both the hydrophilicity of carbonyl compounds and the molecular characteristics of peptides [8]. Studies have shown that glycosylation modification has a great impact on the structure, function, and application of peptides [9]. Covalent binding of peptides to sugars generates new products, leading to changes in the molecular weight, surface charge, and structure of the peptides. Due to the introduction of the hydrophilic group of sugar, the functional characteristics and biological activity of peptides can be significantly improved, which expands their application range [10,11,12].

The Maillard glycosylation reaction is one of the main ways to modify food protein, which is widely used in the food industry. By using some stages of the Maillard reaction, the direct combination of peptides and sugars can be realized under the premise of avoiding the formation of melanosis-like substances as far as possible, so as to improve the biological activity and processing function characteristics of peptides [13]. Compared with the antioxidant peptides, the peptide products produced by glycosylation showed better biological activities. Kang et al. [14] prepared the product of forest frog fragment collagen peptide-xylose, and the results showed that glycosylation could enhance the antioxidant activity of forest frog fragment collagen peptide. Some researchers have also found that glycosylation modification can significantly improve the antioxidant activity of hydrolysates [15,16]. In addition, Gu et al. [17] showed that the amount of glycosylation products was positively correlated with the antioxidant activity, that is, with the increase in the amount of glycosylation products, the antioxidant activity also increased significantly. Feng et al. [18] prepared the Maillard reaction products (MRPs) of zein peptides and obtained higher content of α-dicarbonyl compounds, which had a significant positive effect on DPPH free radical scavenging activity. In addition, the presence of sugar may promote the body’s glucose metabolism pathway, and cooperate with peptides to alleviate oxidative stress. It can be concluded that modification of peptides through the Maillard glycosylation pathway is an effective way to improve their biological activity.

Heavy drinking or long-term drinking can cause alcoholic hepatitis, and in severe cases, liver fibrosis and even liver cancer and other liver damage diseases [19]. Studies have shown that oxidative stress and inflammatory reaction are the main causes of alcoholic liver injury [20]. The intake of substances with antioxidant activity can improve the antioxidant capacity of the body and significantly improve alcoholic liver injury [21]. The zebrafish line CZ320/CZ321 is a transgenic zebrafish with green fluorescent protein expression driven by the zebrafish Apo-14 promoter sequence. The Apo-14 promoter-driven green fluorescent protein (GFP) could be continuously expressed in hepatocytes in the liver [22]. Therefore, the dynamic process of liver organogenesis can be observed and recorded in detail by observing the changes in liver fluorescence intensity, which is suitable for the study of alcoholic liver injury [22]. Wang et al. [23] established a zebrafish H_2_O_2_ injury model to study the effect of polysaccharide on zebrafish embryonic morphology and the protective effect on cells under oxidative stress and showed that polysaccharide had good antioxidant activity. Cao et al. [24] established a zebrafish fluoride injury model and showed that sesamin can greatly reduce oxidative stress and fluoride damage in zebrafish liver, indicating that sesamin has a certain therapeutic effect on liver health and prevention of fluorosis damage. Therefore, zebrafish as a model organism is feasible to study the natural active substances against alcohol-induced liver injury.

In this study, we prepared PPH-M by the Maillard glycosylation from pea protein hydrolyzed by Alcalase, and analyzed its structural and functional properties. On this basis, the zebrafish model of alcoholic liver injury was constructed, and the mechanism of PPH-M against alcoholic liver injury was revealed by analyzing biochemical indicators, pathological sections, and related mRNA expression levels. This study provided new ideas for high-value utilization and new product development of pea protein and a theoretical basis for enriching the variety of pea protein functional foods.

## 2. Materials and Methods

### 2.1. Materials and Chemicals

Pea protein powder was purchased from Shandong Jianyuan Food Co., Ltd., and protein content was 72.52% (Taian, China). Alcalase (23,000 U/mL) was purchased from Novo Nordisk (Bagsvaerd, Denmark). D-xylose was bought from Tianjin Kemiou Chemical Reagent Co., Ltd. (Tianjin, China). DPPH was purchased from Sangon Biotech. Co., Ltd. (Shanghai, China). Zebrafish strain CZ320/CZ321 was purchased from Chinese Zebrafish Resource Center (Wuhan, China). The RNA extraction kit was purchased from Omega Biotech Co., Ltd. (Norcross, GA, USA). The cDNA kit was bought from Takara Biomedical Co., Ltd. (Beijing, China). qRT-PCR SYBR Green Premix was obtained from Thermo Fisher Scientific (Waltham, MA, USA).

### 2.2. Preparation of PPH and PPH-M

The substrate concentration of the 5% (*w*/*v*) pea protein solution was prepared, and the pH was adjusted to 8.5. The solution was digested with Alcalase at 50 °C for 2 h, and the amount of enzyme was 1000 U/g. After the reaction, the enzyme was inactivated by boiling in a water bath for 10 min, cooled to room temperature, centrifuged at 4000 r/min for 15 min, and the supernatant was lyophilized to obtain PPH.

Glycosylation modification of PPH was performed in a water bath with continuous stirring. A certain concentration of PPH solution was prepared, D-xylose was added according to the appropriate mass ratio of glycopeptide, and the pH value of the solution was adjusted by stirring at a constant speed at an appropriate temperature. The changes in the following parameters during glycosylation were as follows: pH of glycosylation (7.0, 7.5, 8.0, 8.5, 9.0), reaction time (1 h, 2 h, 3 h, 4 h, 5 h), reaction temperature (70 °C, 75 °C, 80 °C, 85 °C, 90 °C), glycopeptide mass ratio (1:3, 1:2, 1:1, 2:1, 3:1), and PPH concentration (1%, 2%, 3%, 4%, 5%). The solution was cooled and dialyzed with a 200 Da dialysis bag for 48 h at 4 °C, and the pea glycopeptides (PPH-M) were obtained after lyophilization.

### 2.3. Structural Characterization of PPH and PPH-M

#### 2.3.1. Fourier Transform Infrared Spectroscopy

According to the method of Song et al. [25], the KBr was used for pretreatment. The lyophilized samples (1 mg) of PPH and PPH-M were weighed, mixed, and ground with a certain amount of KBr powder, pressed into solid films, scanned 32 times in the band of 4000–400 cm^−1^ at a resolution of 4 cm^−1^, and the infrared spectra of the samples were collected.

#### 2.3.2. UV Spectroscopic Determination

According to the method of Zheng et al. [26], slight changes were made. A sample solution of 2 mg/mL was prepared with phosphate buffer solution, injected into a quartz cuvette, and scanned with a U-2900 UV spectrophotometer in the range of 245 to 400 nm to compare the changes in the UV absorption intensity.

#### 2.3.3. Determination of Molecular Weight Distribution

The molecular weight distribution of PPH and PPH-M was determined using a gel column, Superdex Peptide 10/300 GL. The samples were dissolved in 0.02 mol/L pH 7.0 phosphate buffer (containing 0.15 mol/L NaCl), a 2 mg/mL (protein concentration) solution was prepared, centrifuged for 10 min at 4 °C at 10,000 r/min, and the supernatant was filtered through a 0.22 μm microporous filter membrane before loading. Blue Dextran 2000 was used to determine the volume of external water. The standard proteins were aprotinin (6511 Da), bacitracin (1422 Da), oxidized glutathione (612 Da), and reduced glutathione (307 Da), and the effective partition coefficient (Kaw) was used as the abscissa. The logarithm of relative molecular mass of standard protein (log Mr) was the standard curve on the ordinate, and the relative molecular mass of protein was calculated. The standard curve was y = −3.4524x + 4.6936, R^2^ = 0.9878.

#### 2.3.4. Scanning Electron Microscopy

Referring to the method of Liu et al. [27], the sample was fixed on the testing table with conductive double-sided glue. The dried samples were sprinkled on double-sided glue and gently placed on the testing table and excess powder was blown off. After spraying gold under vacuum, the samples were placed under a KYKY-EM 3200 scanning electron microscope (Beijing, China) and scanned under accelerating voltage.

#### 2.3.5. Zeta Potential and Particle Size Determination

The 1 mg/mL PPH and PPH-M solutions were prepared with deionized water, respectively. The zeta potential and average particle size of the sample solutions were determined by the Nano Malvern laser particle size meter. The determination time was set as 120 s, and the DTS1070 dish was used for repeated determination 20 times at room temperature (25 °C).

#### 2.3.6. Determination of Amino Acid Composition

According to the protein content of the sample, about 0.06 g of the sample was weighed and loaded into the ampoule tube, and 9 mL of 6 mol/L HCl was added. After nitrogen blowing for 10 min, the tube was sealed. Acid hydrolysis was carried out in an oven at 110 °C for 24 h. After cooling, 8.5 mL of 6 mol/L NaOH was added, the pH was adjusted to 2.2, filtered, and the solution was poured into a 100 mL volumetric flask, and the volumetric flask was filled with citrate buffer (pH 2.2). The solution was analyzed with an automatic amino acid analyzer.

### 2.4. PPH-M Antagonized Alcoholic Liver Injury in Zebrafish

#### 2.4.1. Zebrafish Feeding and Embryo Collection

Zebrafish were maintained in the Zebrafish Laboratory, School of Food and Bioengineering, Qiqihar University. The Zebrafish experiment was approved by the Animal Ethics Committee of the College of Food and Bioengineering, Qiqihar University. The Zebrafish were maintained by the Qiqihar University Regulations on Animal Experimentation (Approval No. 2022-005). Adult zebrafish were reared in a specific culture system with a water temperature of 28 °C, a pH of 6.5–7.5, a conductivity of 450–550 μS, a salt concentration of 0.03–0.04%, and a light/dark cycle of 14/10 h. Zebrafish adults were fed with brine shrimp once a day, morning and evening.

At 3 months of age, zebrafish reach sexual maturity and can be used for mating. The day before spawning, the adult zebrafish were fed for half an hour in the evening, and healthy zebrafish were selected and placed in the oviposition box according to the ratio of 1:1 or 1:2. The female and male were separated by a baffle, and the fish chamber was kept in the dark. After 30 min, the embryos were collected in the culture dish, washed with system water several times, sterilized with 0.1% methylene blue, and then incubated in a 28.5 °C constant temperature incubator under light control for later use.

#### 2.4.2. Establishment of Alcohol Injury Model in Zebrafish

**Figure 1:**
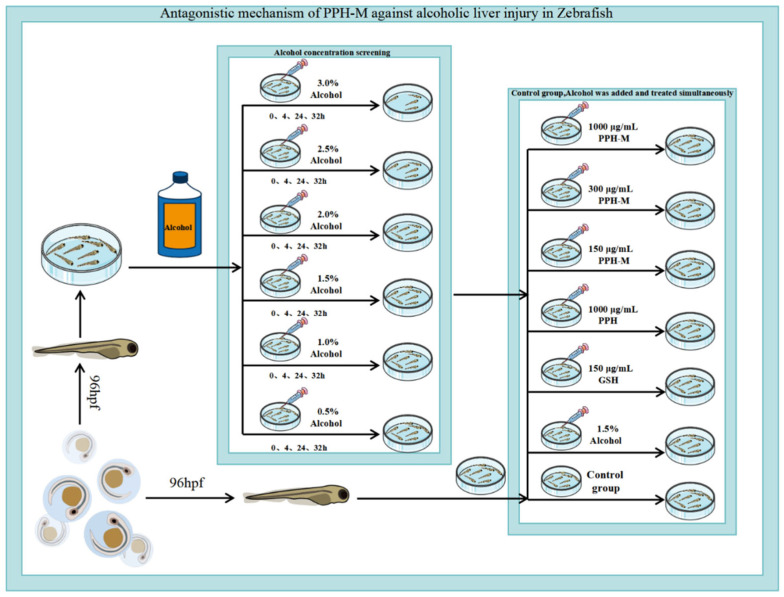
Establishment of alcoholic liver injury model in zebrafish and animal experimental design of antagonistic effect of PPH-M on alcohol-induced liver injury in zebrafish.

The liver of the fertilized zebrafish was mature at 96 post-fertilization hours (hpf). Therefore, 630 zebrafishes with normal liver development were selected to develop embryos to 96 hpf. As shown in Figure 1, they were randomly divided into the control group, alcohol concentration 0.5%, 1.0%, 1.5%, 2.0%, 2.5%, and 3.0% (*v*/*v*) groups, which were placed in six-well plates, and each treatment group had three parallel wells, 30 zebrafishes in each well. The control group was cultured with water from the zebrafish culture system, and the alcohol treatment group was cultured with a series of alcohol solutions prepared from the zebrafish culture system water. Each well was marked for time and group, and the six-well plate was capped and cultured in a constant temperature incubator at 28 °C. After 32 h, the morphology and survival of juveniles were observed and recorded, and samples were collected to determine the alcohol concentration and time for establishing the model.

#### 2.4.3. Grouping and Drug Administration

A total of 450 zebrafishes with normal liver development at 96 hpf were randomly divided into 250 μg/mL, 500 μg/mL, 1000 μg/mL, 2000 μg/mL, and 3000 μg/mL PPH-M treatment groups, which were placed in six-well plates with three parallel wells in each treatment group. There were 30 zebrafishes per well. Six-well plates were covered and cultured in a constant temperature incubator at 28 °C. After a certain period of time, the morphology and survival of zebrafish were observed and recorded to determine the maximum drug concentration of zebrafish.

The zebrafishes were divided into seven groups (Figure 1): control group, model group, positive (GSH, 150 μg/mL) group, PPH group (1000 μg/mL), low-dose PPH-M (150 μg/mL), medium-dose PPH-M (300 μg/mL), and high-dose (1000 μg/mL) PPH-M group. The corresponding samples were added, respectively, and 1.5% alcohol solution was added to the culture for 24 h and then incubated in a constant temperature incubator at 28 °C. The above experiments were conducted in each group three times in parallel, and each parallel had 30 zebrafishes.

#### 2.4.4. Morphological Observation of Zebrafish Liver

After 24 h of treatment according to Section 2.4.3, the fluorescence and morphological changes in zebrafish liver were observed under a fluorescence microscope and stereomicroscope.

#### 2.4.5. Pathological Evaluation of Zebrafish Liver Tissue

After drug treatment, the intact zebrafish larvae were fixed with 4% paraformaldehyde overnight, bound with paraffin, and then sliced. The paraffin sections are first immersed in xylene solution for 10 min to remove the paraffin, and then placed in a series of ethanol solutions for 5 min each (100%, 95%, 85%, and 75% ethanol) to rehydrate. The slices were dried and finally stained with hematoxylin-eosin (HE), and the liver tissue was photographed for observation.

#### 2.4.6. Detection of Related Biochemical Indexes of Zebrafish Liver

Following the administration treatment, the livers of zebrafish were separated under a microscope, transferred to EP tubes, washed with PBS solution, and homogenized for 3 min on ice using a cell tissue grinder to prepare homogenate. All tests should be carried out in an ice bath. The homogenate was centrifuged at 10,000 r/min at 4 °C for 5 min, and the supernatant was removed. The relevant indicators were detected according to the instructions of the ELISA kit (Keqiao Co. Ltd., Shanghai, China), and each group was repeated three times.

#### 2.4.7. Detection of Related Genes by qRT-PCR

After the experiment in accordance with Section 2.4.3, total RNA from juvenile liver was extracted for reverse transcription, and the resulting cDNA was used for RT-PCR amplification. The expression levels of antioxidant-related genes kelch-like ECH-associated protein 1 (Keap1), nuclear respirator factor 2 (Nrf2), superoxide dismutase (SOD) and catalase (CAT), and glutathione synthesis-related genes, glutathione s-transferase kappa 1 (Gstk1), glutathione reductase (Gsr), glutathione peroxidase I (Gpxla), and isocitrate dehydrogenase 1 (Idh1) in zebrafish were detected. The qRT-PCR system and cycle number are shown in Table 1. The gene β-actin was set as the reference gene to calculate mRNA expression, and the relevant primer sequences are shown in Table 2.

The reaction system reaction solution was prepared, carried out in an ice bath, accurately added to a 96-well plate, centrifuged until there were no bubbles at all, and placed inside the QuantStudio Real-Time Fluorescence Quantitative PCR System. The amplification conditions were as follows: 50 °C for 2 min, followed by preheating at 95 °C for 2 min. A total of 45 cycles were performed at 95 °C for 15 s, 60 °C for 15 s, and 72 °C for 1 min, followed by 95 °C for 15 s and 60 °C for 1 min.

### 2.5. Statistical Analysis

All the data of this experiment were analyzed by IBM SPSS Statistics 26 software, and are expressed as mean ± standard deviations (SDs). All data were analyzed by the Shapiro–Wilk test to check for normality of data distribution, and then statistical comparisons were performed using one-way analysis of variance (ANOVA) and Duncan’s test was used as a post hoc test following ANOVA. The data were plotted by Origin 2019 software.

## 3. Results and Discussion

### 3.1. Antioxidant Activity Analysis of PPH and PPH-M In Vitro

Reducing sugar is one of the important factors affecting Maillard glycosylation, and the type of reducing sugar has a great influence on the degree of glycosylation reaction and the biological activity of products. In our previous study, PPH was glycosylated with D-galactose, D-glucose, D-fructose, D-xylose, D-maltose, and D-lactose, and it was found that the antioxidant activity of glycosylated products (PPH-M) prepared by D-xylose was significantly improved. Previous studies have reported that the type of reducing sugar affects the antioxidant activity of glycosylated products in the order of aldopentoses > aldohexose > ketohexose > disaccharide [28]. Zhan et al. [29] studied the effects of glucose, fructose, and D-xylose on the antioxidant properties of duck liver protein glycosylation products, and the results showed that the duck liver protein glycosylation products combined with D-xylose had the best antioxidant properties. Therefore, we selected D-xylose as the best sugar source to optimize the preparation conditions of the glycosylation products.

In order to maximize the binding degree of D-xylose to PPH, we optimized the glycosylation modification conditions based on the DPPH free radical scavenging rate and the amount of sugar bound. The optimal reaction conditions were determined as follows: pH 9.0, reaction time 3 h, reaction temperature 90 °C, glycopeptide mass ratio 2:1, and PPH concentration 3%. Under this reaction condition, the DPPH free radical scavenging rate of PPH-M (protein concentration 2 mg/mL) was 88.92%, and the amount of sugar bound was 135.64 mg/g. The DPPH free radical scavenging rate of PPH-M prepared in this study was significantly higher than that of glycopeptides such as carboxymethyl chitosan-silk fibroin peptide (CMC-SP) and advanced glycation end products of glutaminase hydrolysates (AGEs) reported [30,31]. Chen et al. [32] also found that the D-xylose glycosylated snapper peptides had better antioxidant activity. Compared with PPH, the DPPH radical scavenging rate of PPH-M was increased by 50.37% at the same protein concentration. The significant improvement of PPH-M antioxidant activity is closely related to the introduction of D-xylose. The combination of D-xylose makes PPH-M have more hydrogen donors, which can effectively combine with DPPH free radicals to form a stable molecular structure [33].

### 3.2. Conjugation of Pea Peptides and D-Xylose Was Confirmed by FTIR

FTIR can effectively detect the changes in protein functional groups, among which the information in the infrared spectrum is mainly the frequency doubling and fusion of the inter-atomic vibration inside the molecule [34]. The main spectral features of the absorption of commonly used protein structures are the amide I band at 1700–1600 cm^−1^ (stretching vibration of C=O on the peptide chain, N-H stretching) and the amide II band at 1600–1500 cm^−1^ (C-N stretching and N-H coiling) [35].

**Figure 2:**
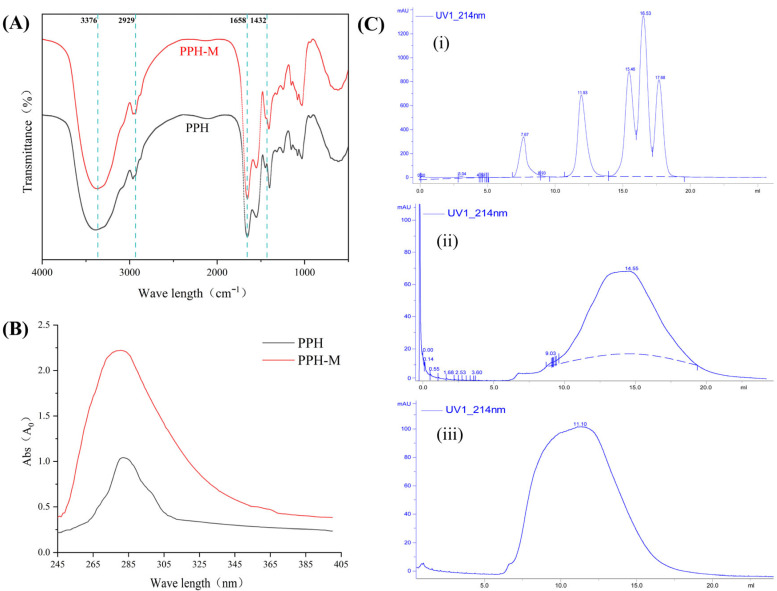
Structural characterization of PPH and PPH-M. (**A**) The FTIR of PPH and PPH-M. (**B**) UV spectrogram of PPH and PPH-M. (**C**) Molecular weight distribution elution of PPH and PPH-M, (**i**) standard protein, (**ii**) PPH, and (**iii**) PPH-M.

As shown in Figure 2A, the characteristic absorption peak of PPH appears at 3376 cm^−1^, which is attributed to the stretching vibration of the N-H bond, while the absorption peak of PPH-M at this point is redshifted from 3399 cm^−1^ to 3376 cm^−1^, reflecting the change in its spatial structure after the glycosylation modification of PPH [36]. Compared with PPH, PPH-M showed a short and sharp absorption peak at 2929 cm^−1^, which was attributed to the C-H stretching vibration, probably due to the C-H stretching vibration in the sugar molecule after glycosylation modification. An absorption peak appeared at 1642 cm^−1^, belonging to the amide I band (1700–1600 cm^−1^), indicating that the glycosylation modification of PPH produced strong C=O stretching and C-N stretching vibrations. Compared with PPH, the absorption peak of PPH-M disappeared at 1432 cm^−1^ and became wider at 1409 cm^−1^, which may be caused by the inward bending vibration of C-H and the stretching vibration of N-H of the primary amide [37]. In the process of glycosylation, the carbonyl group in D-xylose is linked to the amino group of PPH by a covalent bond, which causes deformation vibration in the terminal sugar ring. At the same time, the new C-N covalent bond is formed in the product of glycosylation modification, and the stretching vibration absorption intensity is increased. All these confirm the occurrence of glycosylation, and it is found that C=O and N-H both produce deformation vibration. These results indicated that PPH formed a covalent bond during the glycosylation modification reaction, which further confirmed the reaction between PPH and D-xylose.

### 3.3. UV Spectrum Analysis

UV spectrum is a common method for protein conformation research, in which the change in absorption peak in the near ultraviolet region is derived from aromatic compounds, which can reflect the change in the protein side chain and disulfide bond [38]. The glycosylation of PPH was confirmed by measuring and analyzing the UV absorption spectra of PPH and PPH-M.

According to Figure 2B, PPH and PPH-M were scanned in the full wavelength of 245–400 nm. By comparing the UV spectra of PPH and PPH-M, it was found that there was a characteristic peak at the wavelength of 280 nm, where the absorption of UV light was mainly due to the conjugated double bond in the side chain of aromatic amino acids. There is no obvious red shift or blue shift in the maximum absorption peak of PPH-M, but the UV absorption intensity of the characteristic peak is increased, which may be due to the change in the structure of PPH caused by glycosylation, which gradually migrates some aromatic amino acids from the inside of the molecule to the outside [39]. At the same time, in the process of Maillard glycosylation, small molecules such as aldehydes, ketones, and peptides polymerize to form melanoid, which will also cause the phenomenon of increased absorbance [40]. In summary, the results of UV spectra indicated the occurrence of PPH glycosylation.

### 3.4. Molecular Weight Distribution of PPH-M Becomes Larger

Determination of the molecular weight distribution of PPH and PPH-M by gel chromatography can assist in the analysis of the occurrence of PPH glycosylation modification. As shown in Figure 2C, 44.27% of the components in PPH have a molecular weight less than 1.6 kDa, 44.61% of the components have a molecular weight between 1.6 kDa and 20.0 kDa, and 11.12% of the components have a molecular weight greater than 20.0 kDa. After glycosylation, the fraction of PPH-M with a molecular weight less than 1.6 kDa was 3.97%, the fraction with a molecular weight between 1.6 kDa and 20.0 kDa was 77.26%, and the fraction with a molecular weight more than 20.0 kDa was 18.77% (Table 3). It can be seen that the molecular weight of PPH was significantly increased after glycosylation modification, and the proportion of macromolecular components was significantly increased. The reason may be that D-xylose was bound to the free amino group on the PPH chain by glycosylation, and D-xylose was grafted to the peptide chain, resulting in the increase in the molecular weight of PPH [41]. He et al. [42] measured the molecular weight of zein hydrolysate and its glycosylation products by sodium dodecyl sulfate polyacrylamide gel electrophoresis. The results showed that compared with zein hydrolysate, the high molecular weight band of glycosylation products of zein hydrolysate was significantly increased, and the amount of macromolecular substances was increased with the extension of reaction time.

### 3.5. SEM Analysis

SEM analysis is a means of analyzing the microscopic morphology of objects and observing structural features, which can intuitively show the structure and morphology of substances [43]. The surface microstructure changes in PPH and PPH-M were analyzed by SEM.

**Figure 3:**
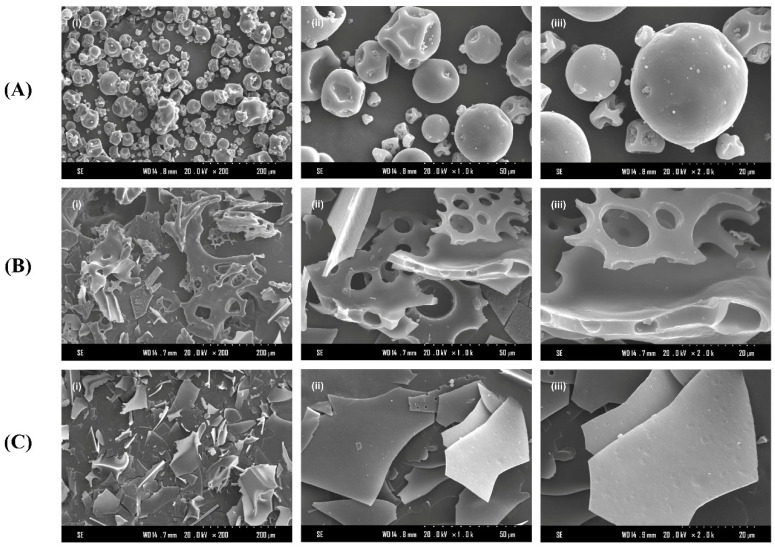
SEM of (**A**) pea protein, (**B**) PPH, and (**C**) PPH-M. (**i**) ×200, (**ii**) ×1000, and (**iii**) ×2000.

As shown in Figure 3, the microstructure of PPH and PPH-M is obviously different. The microstructure of pea protein aggregates mainly presents an irregular form close to a spherical shape. The spherical shape has a large diameter, most of which has a smooth surface and relatively concentrated distribution, and some particles have smaller spherical structures adhered to the surface, which is consistent with the observation of Khan et al. [44]. After enzymatic hydrolysis, PPH aggregates showed that the original spherical structure was destroyed and formed a large sheet with a certain thickness with gaps and a porous structure. This may be due to the change in the protein structure of pea protein after alkaline protease treatment, which made the original spherical aggregate structure expand into a sheet. In contrast, when PPH was modified by glycosylation, the aggregates exhibited a compact sheet structure with a smooth surface, and the pore pattern disappeared. Due to the introduction of new charged groups, the interaction force between the glycosylation product aggregates is changed, and a more uniform morphology is formed. Zha et al. [45] found that the product structure with a similar flat surface was formed when they studied the coupling product of pea protein hydrolysate and gum arabic (GA). This result also again indicates that the glycosylation reaction occurred and that the structure of PPH changed significantly.

### 3.6. Zeta Potential and Particle Size Analysis

Zeta potential plays a leading role in the droplet instability, and the absolute value of zeta potential can represent the strength of electrostatic interaction. When the absolute value of zeta potential is between 0 and 15 mV, the sample solution is more prone to flocculation, aggregation, and stratification. When the absolute value of zeta potential is greater than 15 mV, the solution is more stable and less likely to aggregate. The more obvious the electrostatic repulsion between particles, the longer the distance between particles [46].

**Figure 4:**
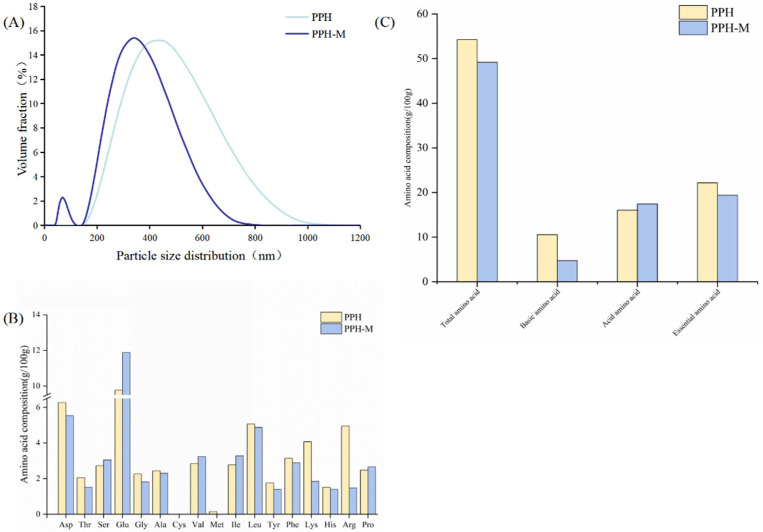
(**A**) Particle size of PPH and PPH-M. (**B**,**C**) Amino acid composition analysis of PPH and PPH-M.

The absolute value of PPH increased from 11.50 mV to 21.37 mV after D-xylose modification (Table 4), which may be due to the structural changes in PPH after glycosylation modification, and the internal charge of the PPH-M migrated to the surface, leading to the increase in the charge amount, that is, the potential value showed a decreasing trend [47]. As shown in Figure 4A, the particle size distribution of PPH becomes smaller after glycosylation modification. The average particle size of PPH is 416.2 nm, and the average particle size of PPH-M is 314.7 nm. Although the molecular weight of PPH-M is increased, the introduction of a hydroxyl group in D-xylose leads to the increase in the same electrical charge on its surface, and the steric repulsion of PPH-M is enhanced, which inhibits the binding tendency and reduces the formation of PPH-M aggregates, resulting in the decrease in the particle size of PPH-M. This is consistent with the findings of Wang et al. [40].

### 3.7. Amino Acid Composition

The amino acid composition of PPH and PPH-M was analyzed by an automatic amino acid analyzer. Figure 4B,C show that the total amino acid content of PPH-M decreased from 54.24 g/100 g to 49.19 g/100 g, a reduction of 9.31%. The basic amino acids decreased from 10.53 g/100 g to 4.73 g/100 g, with a decrease of 55.08%. The relative content of Lys, His, and Arg in the basic amino acids decreased from 4.07, 1.51, and 4.95 g/100 g to 1.84, 1.41, and 1.48 g/100 g, respectively, with a reduction of 54.8%, 6.6%, and 70.1%, respectively. The content of essential amino acids was reduced by 12.59%. On the one hand, Lys, His, and Arg are basic amino acids, and there are free amino groups on the side chain, which are consumed with D-xylose to participate in glycosylation modification. On the other hand, due to the successful grafting of D-xylose into PPH, the D-xylose component is increased in unit mass, so the proportion of the total amino acid content decreases. In addition, although the content of essential amino acids in PPH-M is slightly reduced, the amino acid composition of PPH and PPH-M is unique, and the content of other essential amino acids in PPH-M is relatively balanced, and PPH-M is still a good source of essential amino acids [48,49].

### 3.8. Screening of Alcohol Concentration in Zebrafish Liver Injury

**Figure 5:**
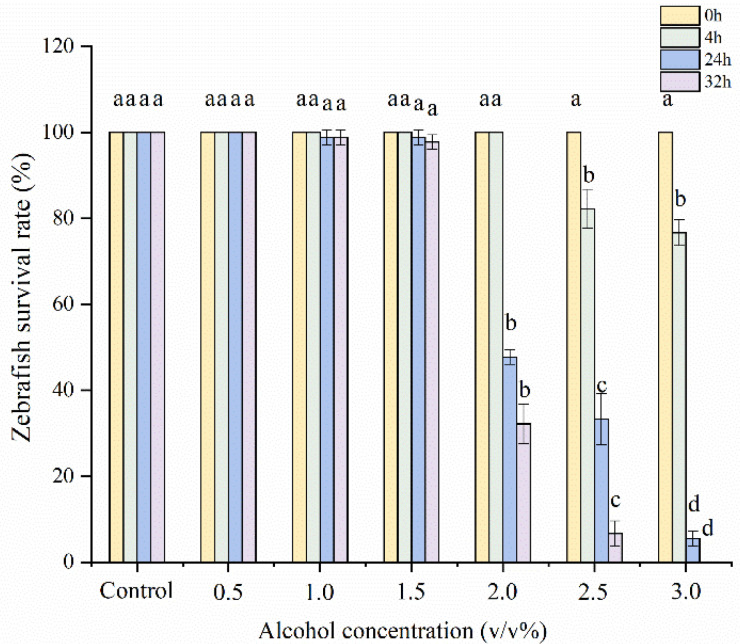
Effect of alcohol concentration on survival of zebrafish. Different lowercase letters represent significant differences within groups (*p* < 0.05).

**Figure 6:**
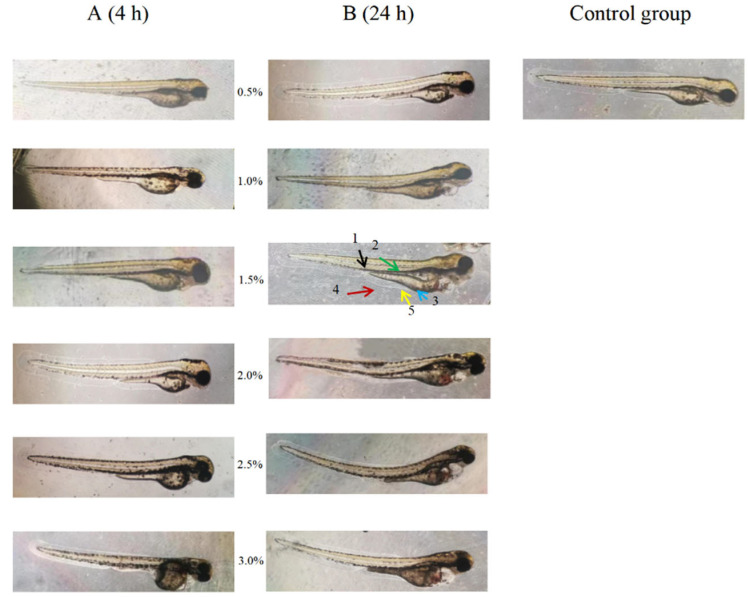
Effects of alcohol concentration and time on zebrafish morphology: (**A**) 4 h and (**B**) 24 h. Black arrow represents spine, green arrow represents swim bladder, blue arrow represents pericardium, red arrow represents yolk sac, and yellow arrow represents luminal hemorrhage.

In order to further explore the mechanism of PPH-M in antagonizing alcoholic liver injury in zebrafish, an alcohol-induced zebrafish liver injury model was established. As shown in Figure 5, after the zebrafish developed to 96 hpf, they were treated with different alcohol concentrations for 32 h, and the effects of alcohol treatment time and concentration on the survival rate of zebrafish were negatively correlated. When treated with alcohol for 4 h, the survival rates of zebrafish treated with 2.5% alcohol and 3.0% alcohol were significantly decreased, and the survival rates were 82.22 ± 4.55% and 76.67 ± 2.98%, respectively. All zebrafish treated with other alcohol concentrations survived. When the treatment time was 24 h, the mortality rate was not significantly increased when the alcohol concentration was lower than 1.5%. However, concentrations of 2.0% and above resulted in the death of more than 50% of zebrafish. After 32 h of the experiment, the survival rate of zebrafish treated with 1.5% alcohol concentration was relatively stable compared with that at 24 h, with a survival rate of 97.78 ± 1.72%. This observation is consistent with the conclusion of the study by Tsedensodnom et al. [20]. At the same time, the morphological changes in zebrafish under different concentrations of alcohol were observed under a microscope, and the results showed that in the same action time, high alcohol concentration showed more obvious toxic effects (Figure 6). Deformities, spinal curvature, pericardial enlargement, yolk sac edema, and hemorrhage were observed in zebrafish treated with 1.5% alcohol for 24 h. The results showed that the developmental toxicity of zebrafish treated with 1.5% alcohol for 24 h after injury could be affected, and this modeling condition had a liver damage effect on zebrafish.

**Figure 7:**
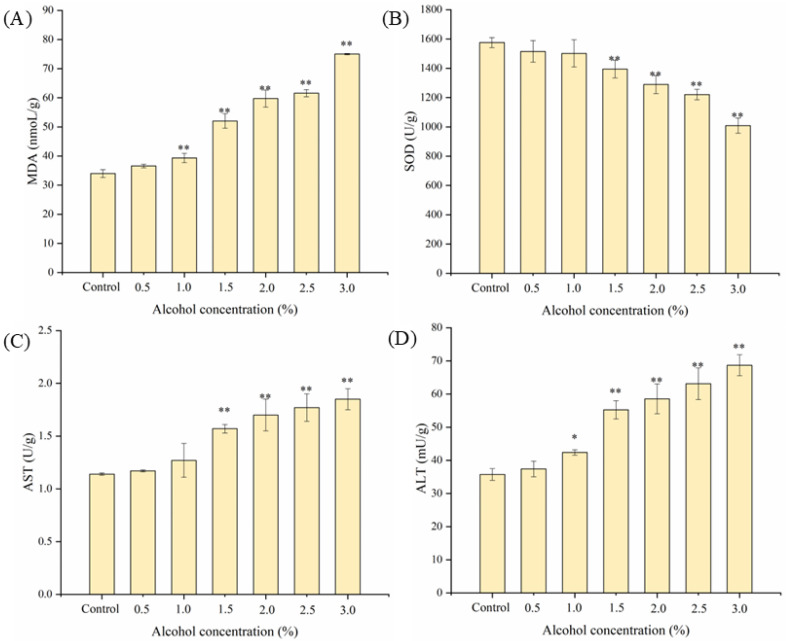
Effects of alcohol concentration on (**A**) MDA, (**B**) SOD, (**C**) AST, and (**D**) ALT in zebrafish. Compared with control group, * represents significant differences (*p* < 0.05) and ** represents extremely significant differences (*p* < 0.01).

The changes in physiological indicators related to oxidative stress in zebrafish were further analyzed. As shown in Figure 7, compared with the control group, with the increase in alcohol concentration, the content of malondialdehyde (MDA) increased gradually, the activity of superoxide dismutase (SOD) decreased gradually, and the activities of aspartate aminotransferase (AST) and alanine aminotransferase (ALT) increased gradually, showing extremely significant differences (*p* < 0.01). It was found that when the liver was damaged by alcohol, it would induce a severe oxidative stress response in the liver [21], and the biochemical indexes related to oxidation and antioxidation in the body would change significantly. After zebrafish liver was treated with 1.5% alcohol for 24 h, MDA content was 52.03 nmol/g, SOD activity was 1394.94 ± 60.24 U/g, AST activity was 1.57 ± 0.04 U/g, and ALT activity was 55.23 ± 2.76 mU/g. Compared with the control group, they increased by 53.16%, decreased by 11.47%, and increased by 37.72% and 54.53%, respectively. This may be due to ethanol-induced severe lipid peroxidation in the liver of zebrafish, resulting in a significant increase in MDA content (*p* < 0.01), and then leading to oxidative stress and liver injury. AST and ALT are important indicators of liver health, and their levels directly reflect the degree of liver cell injury. The excessive consumption of SOD and the excessive production of MDA, AST, and ALT indicated that 1.5% alcohol concentration caused severe oxidative stress in zebrafish liver tissue.

**Figure 8:**
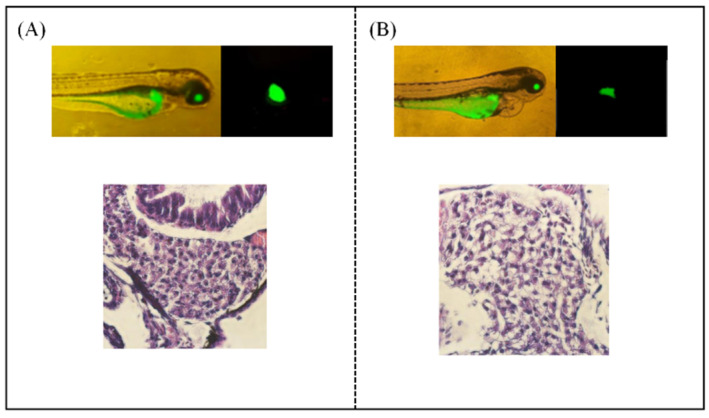
Effect of alcohol on liver injury in zebrafish. Fluorescence intensity of zebrafish liver was observed by fluorescence microscope, and pathology of zebrafish liver tissue: (**A**) the control group and (**B**) the model group.

Zebrafish strain CZ320/CZ321 is a green fluorescent protein driven by the Apo-14 promoter, which can be continuously expressed in adult fish liver primordium and hepatocytes, thus allowing detailed observation of the dynamic process of liver organogenesis. It provides a transgenic tool for the study of liver development and its organogenesis, which can be observed from early endodermal cells to complete liver organ formation [22]. The liver morphology and fluorescence intensity of transgenic zebrafish can be observed in real time by fluorescence microscope. When the zebrafish liver tissue is damaged to a certain extent, the changes in liver morphology can be obviously observed under a fluorescence microscope, and the fluorescence intensity will also weaken. Compared with the control group, the liver of the model group was significantly atrophied and degenerated, and the fluorescence intensity of the liver was significantly reduced. In the control group (Figure 8), the morphology of liver tissue and cell structure were complete and regular, neatly arranged, with clear cell boundaries and uniform and abundant cytoplasm. However, the structure of liver cells in the model group was loose and irregular, with a large number of vacuolar degeneration and steatosis to a certain extent. These results further indicated alcohol-induced liver injury in zebrafish. Taken together, these results suggested that 1.5% alcohol treatment for 24 h was a suitable condition for establishing a zebrafish liver injury model, and the subsequent experiments were carried out based on this model.

### 3.9. Protective Effects of PPH-M on Alcoholic Liver Injury in Zebrafish

**Figure 9:**
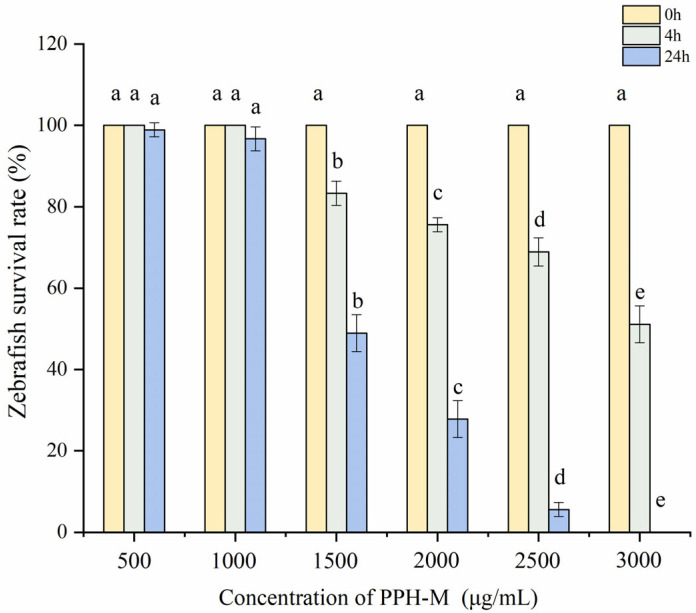
Effect of PPH-M concentration on zebrafish survival. Different lowercase letters represent significant differences within groups (*p* < 0.05).

By analyzing the effect of PPH-M on the survival rate of zebrafish, the appropriate concentration range for the test was determined. Figure 9 shows that the effect of different PPH-M concentrations on the survival rate of zebrafish was concentration dependent. When the concentration of PPH-M was 500 μg/mL, the survival rate of zebrafish after 4 h of intervention was 100%, and the survival rate after 24 h of intervention was 98.89 ± 1.72%, which was not significantly different from that after 4 h. At the same intervention time, with the increase in PPH-M concentration, the survival rate of zebrafish gradually decreased. When the concentration of PPH-M was 3000 μg/mL for 4 h, the survival rate of zebrafish was 51.11 ± 4.55%, and after 24 h of intervention, all the zebrafish died, which may be due to the large amounts of salts contained in PPH-M. When the concentration of PPH-M was 1000 μg/mL, the survival rate of larvae was 96.67 ± 2.98% after 24 h of intervention, which was not significantly different from that of zebrafish at 4 h, indicating that PPH-M had certain safety and did not cause zebrafish death due to PPH-M at this concentration. Therefore, the maximum acting concentration of the PPH-M test was determined to be 1000 μg/mL. Therefore, the PPH-M test concentrations in subsequent experiments were the low-dose group (150 μg/mL), the medium-dose group (300 μg/mL), and the high-dose group (1000 μg/mL), the positive control group (150 μg/mL GSH), and the PPH group (1000 μg/mL).

**Figure 10:**
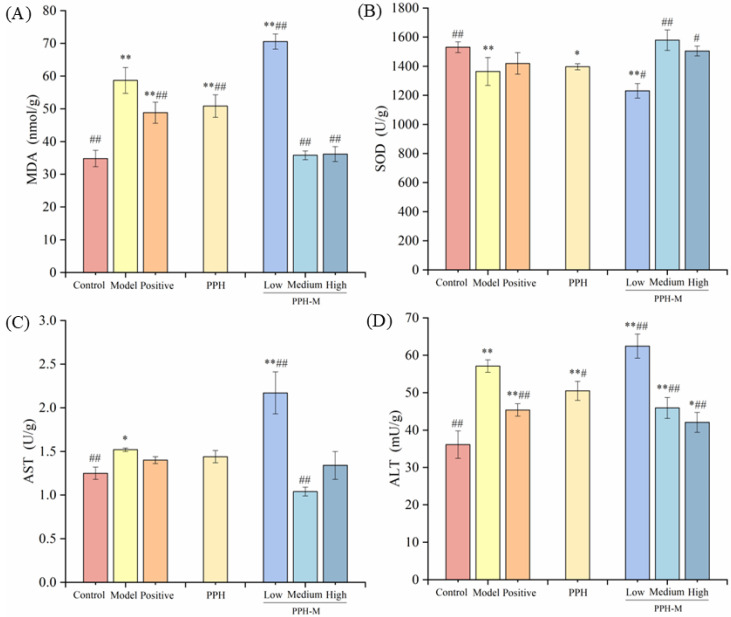
Effects of PPH-M on (**A**) MDA, (**B**) SOD, (**C**) AST, and (**D**) ALT in zebrafish. The concentration of the low-dose group was 150 μg/mL, the medium-dose group was 300 μg/mL, and the high-dose group was 1000 μg/mL. The concentration of the PPH was 1000 μg/mL, and the GSH was 150 μg/mL. Compared with the control group, * represents significant differences (*p* < 0.05) and ** represents extremely significant differences (*p* < 0.01). Compared with the model group, # represents significant differences (*p* < 0.05) and ## represents extremely significant differences (*p* < 0.01).

To analyze the effect of PPH-M on alcohol-induced oxidative stress injury in zebrafish liver, the content of MDA and the activities of SOD, AST, and ALT in zebrafish were measured (Figure 10). As a typical antioxidant enzyme in the body, SOD enzyme can convert superoxide anion into hydrogen peroxide and has a strong ability to remove oxygen free radicals [50]. Compared with the model group, the SOD activity of the experimental group was significantly increased by 15.74% and 10.27%, respectively, under the intervention of medium and high doses of PPH-M (*p* < 0.01), indicating that the intervention of PPH-M could improve the oxidative stress response induced by alcohol and increase the activity of antioxidant enzymes. MDA is a lipid peroxidation product formed under the action of free radicals. MDA content can reflect the antioxidant potential of the body and indirectly reflect the degree of liver damage [51]. Compared with the model group, the content of MDA in the middle- and high-dose PPH-M groups was significantly decreased (*p* < 0.01) by 38.96% and 38.35%, respectively, indicating that the presence of PPH-M inhibited the degree of lipid peroxidation in hepatocytes. AST and ALT are important indicators of liver function, and the changes in AST and ALT content reflect the degree of liver damage. When the body’s liver cells are damaged, AST and ALT will be released into the blood, resulting in the increase in AST and ALT levels [52]. Compared with the model group, the activities of AST and ALT in the middle- and high-dose PPH-M groups were significantly decreased (*p* < 0.05), reduced by 31.58%, 11.84%, 19.59%, 26.35%, respectively. In conclusion, PPH-M at medium and high doses has a certain protective effect on alcohol-induced liver injury in zebrafish, and PPH-M has a better protective effect than PPH.

**Figure 11:**
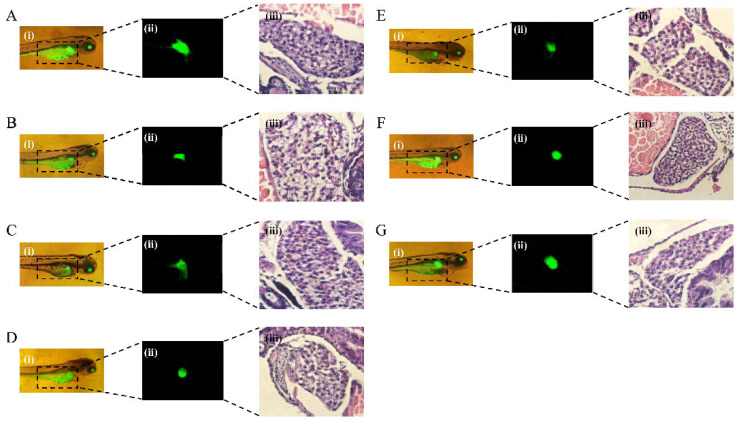
Protective effect of PPH-M intervention on alcohol-induced zebrafish injury, fluorescence images, and pathological sections of liver. (**A**) The control group, (**B**) the model group, (**C**) the positive group (GSH), 150 μg/mL, (**D**) the PPH group, 1000 μg/mL, (**E**) the PPH-M low-dose group, 150 μg/mL, (**F**) the PPH-M medium-dose group, 300 μg/mL, and (**G**) the PPH-M high-dose group, 1000 μg/mL. (**i**) The zebrafish captured by a fluorescence microscope, (**ii**) Fluorescence intensity of zebrafish liver was observed by fluorescence microscope, and (**iii**) pathology of zebrafish liver tissue.

In order to better observe the effect of PPH-M, the fluorescence map and pathological tissue sections of zebrafish liver were observed by fluorescence microscope. According to Figure 11, compared with the control group, the liver fluorescence intensity of the model group was significantly reduced, indicating that alcohol damaged the zebrafish liver and reduced its fluorescence intensity. Compared with the model group, the fluorescence intensity of zebrafish liver in the high-, medium-, and low-dose groups of the PPH-M product was increased, indicating that liver injury was alleviated to a certain extent. The fluorescence intensity and liver size of zebrafish liver in the high-dose PPH-M group were better than those in the positive group and PPH group. Compared with the model group, the fluorescence intensity and size of the zebrafish liver in the PPH group were improved to a certain extent, but there was no significant change. Compared with the PPH, PPH-M can alleviate alcohol-induced liver injury more effectively at the same concentration.

As shown in Figure 11, the liver tissue and cell structure in the control group had complete and regular morphology, neatly arranged, clear cell boundaries, and uniform and abundant cytoplasm. However, in the model group, the structure of liver cells was loose, the arrangement was irregular, and a large number of vacuolar degenerations appeared, indicating that the liver was damaged to a certain extent under the induction of alcohol. Compared with the model group, PPH-M intervention significantly improved the morphology of liver tissue cells. In the low-dose PPH-M group, the liver tissue structure and cell morphology were improved, but the cells were loosely arranged, and there were still a large number of hepatocytes with clear cytoplasm and liver vacuolated morphology. In the middle-dose group of PPH-M, the liver tissue structure and cell morphology were significantly improved, the shape was regular and orderly, the cell boundary was obvious, and the cytoplasm was uniform and abundant, but there were still a small number of liver cells with vacuolated morphology. The high dose of the PPH-M group had the best effect on the improvement of liver cell structure and morphology, which was basically consistent with the control group. The cell morphology was regular, the nucleus was located in the center of the cell, the degree of liver injury was mild, and the protective effect was the most significant. These results indicate that PPH-M has a better protective effect on liver injury in zebrafish.

In conclusion, PPH-M treatment significantly increased SOD activity, decreased MDA content, and decreased AST and ALT activities in the liver tissue of zebrafish. At the same time, compared with the model group, the fluorescence intensity of zebrafish liver was enhanced, and the structure and morphology of liver tissue and cells were improved, among which the high-dose group had the most significant protective effect. These results indicate that PPH-M can significantly improve the antioxidant capacity of zebrafish liver tissue and play a protective role in zebrafish liver tissue.

### 3.10. Effect of PPH-M on Relative Gene Expression of the Keap1/Nrf2 Signaling Pathway in Zebrafish

The kelch-like ECH-associated protein 1 (Keap1)/nuclear respirator factor 2 (Nrf2) signaling pathway is one of the important defense pathways for cell protection and response to oxidative stress, and regulation of this pathway has been confirmed to play an important role in the prevention of oxidative stress-related diseases [53,54]. In order to further explore the regulatory mechanism of PPH-M on the Keap1/Nrf2 signaling pathway and alcohol injury in zebrafish, qRT-PCR was used to analyze the expression levels of related genes in zebrafish liver to further clarify the protective mechanism of PPH-M on alcoholic liver injury in zebrafish.

**Figure 12:**
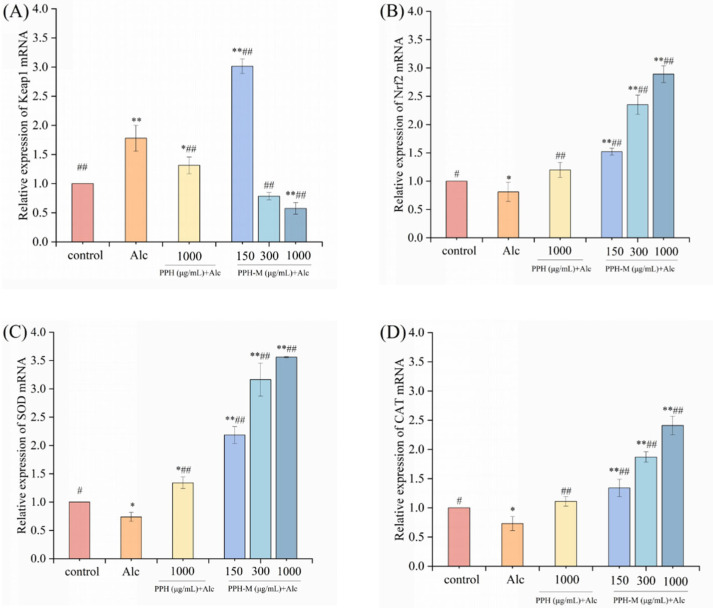
Effect of PPH-M intervention on relative gene expression of the Keap1/Nrf2 signaling pathway in zebrafish. (**A**) Keap1, (**B**) Nrf2, (**C**) SOD, and (**D**) CAT. Compared with the control group, * represents significant differences (*p* < 0.05) and ** represents extremely significant differences (*p* < 0.01). Compared with the model group, # represents significant differences (*p* < 0.05) and ## represents extremely significant differences (*p* < 0.01).

As shown in Figure 12, compared with the control group, the expression level of Keap1 in the model group was significantly up-regulated (*p* < 0.01), increased by 77.90%, while the expression levels of Nrf2, SOD, and CAT genes were significantly down-regulated (*p* < 0.05), decreased by 14.55%, 26.19%, and 15.54%, respectively. The Keap1/Nrf2 signaling pathway is considered to be an important endogenous antioxidant signaling pathway in organisms [53,54]. Nrf2 is an important transcription factor that regulates cellular resistance to oxidative stress. Under normal circumstances, Keap1 binds to Nrf2 to keep it in an inhibited state. Under the influence of external oxidative stress, Nrf2 and Keap1 are uncoupled and transferred into the nucleus, binding to antioxidant response elements and activating the expression of antioxidant enzyme genes and antioxidant proteins regulated by it [55]. It increases the resistance of cells to oxidative stress, thereby playing a protective role. These results indicate that alcohol promotes the overexpression of Keap1, activates the Keap1/Nrf2 signaling pathway, and inhibits the expression of downstream antioxidant enzymes, leading to the increase in ROS in zebrafish and oxidative stress response, which leads to alcoholic liver injury in zebrafish.

Compared with the model group, the expression of Keap1 in the middle- and high-dose PPH-M groups significantly decreased (*p* < 0.01) by 55.95% and 67.70%, respectively, and the expression of Nrf2 significantly increased (*p* < 0.01) by 157.61% and 286.55%, respectively. PPH-M treatment promoted the expression of downstream oxidases SOD and CAT (*p* < 0.01), and the middle-dose group increased the expression of SOD and CAT by 328.48% and 132.45%, and the high-dose group increased the expression of SOD and CAT by 328.54% and 382.37%. The expression of transcription factor Nrf2 and antioxidant enzyme genes in the PPH group did not reach the effect of PPH-M. In the process of alcohol injury, the intervention of PPH-M can significantly prevent the over-activation of the Keap1/Nrf2 signaling pathways, regulate the expression level of antioxidant enzymes, improve the antioxidant capacity of the body, and thus antagonize alcohol-induced liver injury. These results suggest that PPH-M can protect against alcohol-induced liver injury.

The above study found that different concentrations of PPH-M could inhibit the expression of the Keap1 gene, up-regulate the expression of the Nrf2 gene, and up-regulate the expression of SOD and CAT. It plays a protective role in zebrafish liver tissue injury by up-regulating the expression levels of Nrf2 and Keap1/Nrf2 pathway downstream antioxidant enzyme genes.

### 3.11. Effect of PPH-M on Relative Gene Expression of Glutathione Synthesis in Zebrafish

Glutathione (GSH) is one of the most important antioxidants in the body’s antioxidant system. The deficiency of GSH can destroy the redox homeostasis of cells, lead to the accumulation of ROS, and eventually cause cell damage and even death. The relative expression levels of glutathione synthesis-related genes such as Gstk1, Gsr, Gpxla, and Idh1 in zebrafish liver were analyzed by qRT-PCR [56].

Glutathione s-transferase kappa 1 (Gstk1) is a member of the kappa class of the glutathione S-transferase superfamily, which is involved in the regulation of energy production, lipid metabolism, epithelial cell differentiation, and cellular detoxification. The protein encoded by Gstk1 is located in the cell peroxisome and catalyzes the coupling of glutathione with various hydrophobic substances to exert its related biological functions [57,58]. Gstk1 has a specific localization primarily at peroxisomes, where it catalyzes the binding of glutathione to a wide range of hydrophobic subunits. The mitochondrial respiratory chain, mitochondrial and peroxisomal lipid metabolism are major sources of reactive oxygen species such as oxygen radicals, hydroxyl radicals, and hydroperoxides [59].

**Figure 13:**
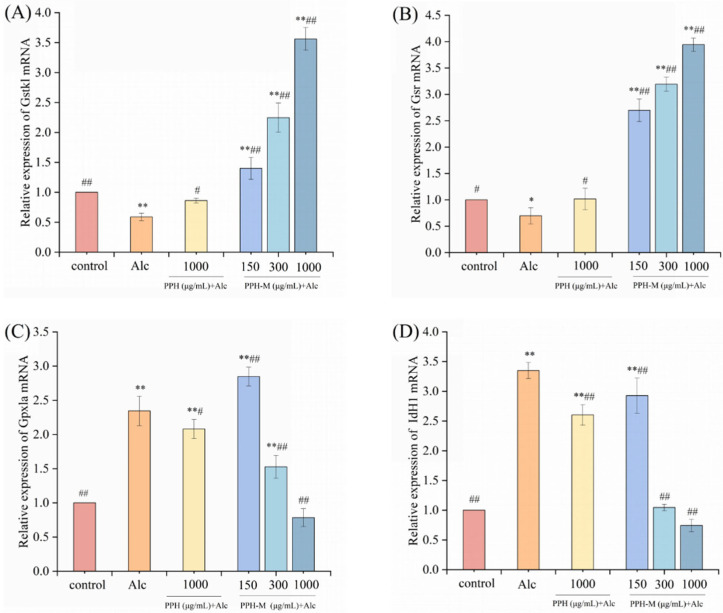
Effect of PPH-M on relative gene expression of the glutathione synthesis in zebrafish. (**A**) Gstk1, (**B**) Gsr, (**C**) Gpxla, and (**D**) IdH1. Compared with the control group, * represents significant differences (*p* < 0.05) and ** represents extremely significant differences (*p* < 0.01). Compared with the model group, # represents significant differences (*p* < 0.05) and ## represents extremely significant differences (*p* < 0.01).

As shown in Figure 13A, compared with the control group, the expression level of Gstk1 mRNA in zebrafish larvae in the model group was significantly down-regulated by 41.18% (*p* < 0.01). Compared with the model group, PPH treatment significantly increased the expression level of Gstk1 (*p* < 0.05) by 13.77%. PPH-M at low, medium, and high doses significantly up-regulated the expression level of Gstk1 (*p* < 0.01), which was superior to the up-regulation ability of PPH.

Glutathione reductase (Gsr) is an enzyme that converts oxidized glutathione to reduced glutathione, which is an important antioxidant in the body [60]. As can be seen from the experimental results in Figure 13B, compared with the control group, the expression of the Gsr gene in the model group was significantly down-regulated (*p* < 0.01), down by 30.27%, indicating that alcohol caused severe oxidative stress in zebrafish and inhibited the expression of Gsr. As a result, the oxidized glutathione in the body cannot be converted into reduced glutathione in time, and the overall antioxidant level decreases. Compared with the model group, the expression level of the Gsr gene was significantly up-regulated in the low-, medium-, and high-dose PPH-M groups (*p* < 0.01), indicating that the intervention of PPH-M can prevent the inhibitory effect of Gsr, promote the field of reduced glutathione, and improve the ability of zebrafish to resist oxidative stress.

Glutathione peroxidase I (Gpxla) is an important peroxidolytic enzyme that widely exists in organisms. It is a kind of glutathione peroxidase subtype I, which can reduce toxic peroxides to non-toxic hydroxyl compounds, thereby protecting the structure and function of cell membranes from interference and damage by oxides [56]. As shown in Figure 13C, compared with the control group, the degree of lipid peroxidation in the liver of the zebrafish model group after exposure to alcohol increased, leading to a significant increase in the Gpxla gene expression level (*p* < 0.01), up 134.46%. These results indicate that the expression of Gpxla is regulated by the redox environment of the body, and the increase in oxidative stress in vivo will increase the expression of Gpxla. Compared with the model group, the low dose of the PPH-M group did not reduce the expression of Gpx1a, while the middle and high doses of the PPH-M groups significantly down-regulated the expression of Gpxla (*p* < 0.01), and the PPH group reduced the expression of Gpxla to a lesser extent.

The isocitrate dehydrogenase 1 (Idh1) gene encodes isocitrate dehydrogenase, which is involved in the catalytic reaction to produce reduced coenzyme II (NADPH). As a donor of reduced hydrogen in vivo, NADPH is involved in the cell’s resistance to oxidative stress on the one hand. On the other hand, it is also involved in the oxidation of unsaturated fatty acids. Studies have shown that increased Idh1 expression can consume more glutathione, thereby aggravating oxidative stress in cells [61]. As shown in Figure 13D, compared with the control group, the expression of Idh1 in the model group was significantly up-regulated (*p* < 0.01), up 234.89%, indicating that alcohol exacerbated oxidative stress in zebrafish. Compared with the model group, the expression level of Idh1 was significantly down-regulated in all PPH-M groups (*p* < 0.01) in a concentration-dependent manner. PPH also decreased the expression level of Idh1, but the ability to down-regulate the gene was not as strong as that of the middle- and high- dose PPH groups. These results indicated that PPH-M could effectively prevent alcohol-induced oxidative stress-induced liver injury in zebrafish.

Taken together, PPH-M can cope with alcohol-induced oxidative stress in zebrafish by regulating the expression levels of related genes and promoting the antioxidant capacity of the body. Compared with PPH, the effect of PPH-M is more significant, showing great application development potential.

## 4. Conclusions

In this study, PPH was covalently combined with D-xylose through the Maillard reaction pathway to prepare PPH-M, which significantly enhanced the antioxidant activity of PPH. FTIR and UV spectra confirmed the binding interaction between them. In addition, the molecular weight distribution and zeta potential analysis further confirmed the successful binding of PPH-M. Finally, PPH-M regulates the Keap1/Nrf2 signaling pathway by increasing the level of antioxidant-related factors and inhibiting the level of oxidative-related factors in the body, thus inhibiting alcoholic liver injury. This study reveals the mechanism of PPH-M antagonizing alcohol-induced liver injury at the molecular level, and this information is of great significance for the development of new functional foods. In future studies, we will further analyze the mechanism of its antagonistic effect on alcoholic liver injury through mice.

## Figures and Tables

**Table 1:** qRT-PCR reaction system.

Ingredient	Content
2 × ChamQ Universal SYBR qPCR Master Mix	10 μL
Forward primer (10 μmol/L)	0.4 μL
Reverse primer (10 μmol/L)	0.4 μL
Template cDNA	1 μL
ddH_2_O	To 20 μL

**Table 2:** Sequences of primers in qRT-PCR.

Gene Abbreviation	Forward Primer	Reverse Primer
β-actin	ACCCCATTGAGCACGGTATT	CTTTGGGATTCAGGGGAGCC
Keap1	GCACTGACCTACACCTTCGC	GCCTTGTAGACCTCGCTCTC
Nrf2	CTCCAAACCTCCGTTCACCA	GTCGTCTACGGGCAGATTGA
CAT	ACATCACGCGCTACTCCAAA	CTGCGAAACCACGAGGATCT
SOD	GGCCAACCGATAGTGTTAGA	CCAGCGTTGCCAGTTTTTAG
Gstk1	TACTTTGGGGTTCCTGTGCG	TCTCCTTCTCTGCTACCGCT
Gsr	CTTGAGTCTTGCCCTAAACGTAG	CTCAGCACCCCTCCTTGTCG
Gpx1a	AGATGTCATTCCTGCACACG	AAGGAGAAGCTTCCTCAGCC
IdH1	ATGCGGTTTGGAGGGTTCAA	ACCTTATCATCGGTGGCGTC

**Table 3:** Molecular weight distribution of PPH and PPH-M.

	<1.6 kDa (%)	1.6 kDa–20 kDa (%)	>20 kDa (%)
PPH	44.27	44.61	11.12
PPH-M	3.97	77.26	18.77

**Table 4:** Zeta potential of the PPH and PPH-M.

Sample	Zeta Potential (mV)
PPH	−11.50 ± 0.92 ^b^
PPH-M	−21.37 ± 0.51 ^a^

Note: Different lowercase letters represent significant differences (*p* < 0.05).

## Data Availability

The original contributions presented in the study are included in the article. Further inquiries can be directed to the corresponding authors.

## References

[1] Ji H., Wang Q., Wang X., Zhang L., Yang P. (2024). Pea protein-inulin conjugate prepared by atmospheric pressure plasma jet combined with glycosylation: Structure and emulsifying properties. Front. Nutr..

[2] Bu F., Nayak G., Bruggeman P., Annor G., Ismail B.P. (2022). Impact of plasma reactive species on the structure and functionality of pea protein isolate. Food Chem..

[3] Lam A.C.Y., Can Karaca A., Tyler R.T., Nickerson M.T. (2016). Pea protein isolates: Structure, extraction, and functionality. Food Rev. Int..

[4] Asledottir T., Vegarud G.E., Picariello G., Mamone G., Lea T.E., Røseth A., Ferranti P., Devold T.G. (2023). Bioactive peptides identified in pea and faba bean after in vitro digestion with human gastrointestinal enzymes. J. Funct. Foods.

[5] Fan H., Wu K., Wu J. (2022). Pea-derived tripeptide LRW fails to reduce blood pressure in spontaneously hypertensive rats due to its low gastrointestinal stability and transepithelial permeability. Food Biosci..

[6] Zhu Y., Zhang H., Wei Y., Cai M., Gu R., Wang Y., Ma Y., Chen L. (2020). Pea-derived peptides, VLP, LLP, VA, and LL, improve insulin resistance in HepG2 cells via activating IRS-1/PI3K/AKT and blocking ROS-mediated p38MAPK signaling. J. Food Biochem..

[7] Ndiaye F., Vuong T., Duarte J., Aluko R.E., Matar C. (2012). Anti-oxidant, anti-inflammatory and immunomodulating properties of an enzymatic protein hydrolysate from yellow field pea seeds. Eur. J. Nutr..

[8] Li M., Huang J., Chen Y., Liu C., Wu X. (2024). Protein from red adzuki bean: Extraction optimization, glycosylation modification and physicochemical properties of glycation products. J. Food Meas. Charact..

[9] Li Y., Lu F., Luo C., Chen Z., Mao J., Shoemaker C., Zhong F. (2009). Functional properties of the Maillard reaction products of rice protein with sugar. Food Chem..

[10] Zhong L., Ma N., Wu Y., Zhao L., Ma G., Pei F., Hu Q. (2019). Characterization and functional evaluation of oat protein isolate-Pleurotus ostreatus β-glucan conjugates formed via Maillard reaction. Food Hydrocoll..

[11] Santagata G., Mallardo S., Fasulo G., Lavermicocca P., Valerio F., Di Biase M., Di Stasio M., Malinconico M., Volpe M.G. (2018). Pectin-honey coating as novel dehydrating bioactive agent for cut fruit: Enhancement of the functional properties of coated dried fruits. Food Chem..

[12] Mulcahy E.M., Park C.W., Drake M., Mulvihill D.M., O’Mahony J.A. (2017). Enhancement of the functional properties of whey protein by conjugation with maltodextrin under dry-heating conditions. Int. J. Dairy Technol..

[13] Wagner K.H., Reichhold S., Koschutnig K., Chériot S., Billaud C. (2007). The potential antimutagenic and antioxidant effects of Maillard reaction products used as “natural antibrowning” agents. Mol. Nutr. Food Res..

[14] Kang N., Song H., Zhang W., Zhao J., Zhang M., Xiong W., Xi C. (2019). Effect of Substrate Ratios and the Species of Sugar on the Antioxidant Activity of Glycosylated Products of the Rana Debris Collagen Peptide. E3S Web Conf..

[15] Liu Q., Li J., Kong B., Jia N., Li P. (2013). Antioxidant capacity of maillard reaction products formed by a porcine plasma protein hydrolysate-sugar model system as related to chemical characteristics. Food Sci. Biotechnol..

[16] Sun Y., Hayakawa S., Puangmanee S., Izumori K. (2006). Chemical properties and antioxidative activity of glycated α-lactalbumin with a rare sugar, d-allose, by Maillard reaction. Food Chem..

[17] Gu F., Kim J.M., Hayat K., Xia S., Feng B., Zhang X. (2009). Characteristics and antioxidant activity of ultrafiltrated Maillard reaction products from a casein–glucose model system. Food Chem..

[18] Feng T., Zhou Y., Wang X., Wang X., Xia S. (2021). α-Dicarbonyl compounds related to antimicrobial and antioxidant activity of maillard reaction products derived from xylose, cysteine and corn peptide hydrolysate. Food Biosci..

[19] Yao F., Abdel-Rahman A.A. (2021). Tetrahydrobiopterin paradoxically mediates cardiac oxidative stress and mitigates ethanol-evoked cardiac dysfunction in conscious female rats. Eur. J. Pharmacol..

[20] Tsedensodnom O., Vacaru A.M., Howarth D.L., Yin C., Sadler K.C. (2013). Ethanol metabolism and oxidative stress are required for unfolded protein response activation and steatosis in alcoholic liver disease. Dis. Models Mech..

[21] Lackner C., Tiniakos D. (2019). Fibrosis and alcohol-related liver disease. J. Hepatol..

[22] Riley B., Wang R., Li Z., Wang Y., Gui J.-F. (2011). An Apo-14 Promoter-Driven Transgenic Zebrafish That Marks Liver Organogenesis. PLoS ONE.

[23] Wang W., Fang S., Xiong Z. (2019). Protective effect of polysaccharide from Ligusticum chuanxiong hort against H2O2-induced toxicity in zebrafish embryo. Carbohydr. Polym..

[24] Cao J., Feng C., Xie L., Li L., Chen J., Yun S., Guo W., Wang T., Wu Y., Meng R. (2020). Sesamin attenuates histological alterations, oxidative stress and expressions of immune-related genes in liver of zebrafish (Danio rerio) exposed to fluoride. Fish Shellfish Immunol..

[25] Song Z., Liu H., Chen L., Chen L., Zhou C., Hong P., Deng C. (2021). Characterization and comparison of collagen extracted from the skin of the Nile tilapia by fermentation and chemical pretreatment. Food Chem..

[26] Zheng Y., Li Y., Ke C., Gao X., Liu Z., Chen J. (2024). Comparison of Structural and Physicochemical Characteristics of Skin Collagen from Chum Salmon (Cold-Water Fish) and Nile Tilapia (Warm-Water Fish). Foods.

[27] Liu R., Shi C., Song Y., Wu T., Zhang M. (2018). Impact of oligomeric procyanidins on wheat gluten microstructure and physicochemical properties. Food Chem..

[28] Jalbout A.F., Shipar M.A.H., Navarro J.L. (2007). Density functional computational studies on ribose and glycine Maillard reaction: Formation of the Amadori rearrangement products in aqueous solution. Food Chem..

[29] Zhan F., Luo J., Sun Y., Hu Y., Fan X., Pan D. (2023). Antioxidant Activity and Cell Protection of Glycosylated Products in Different Reducing Sugar Duck Liver Protein Systems. Foods.

[30] Li H., Ping Y., Niranjan K., Wu Q., Chen Z., Zhang L., Zhao B., Liu K. (2024). Structure, antioxidant properties and AGEs (advanced glycation end products) formation of modified wheat gluten protein after enzymatic hydrolysis and Maillard reaction. J. Food Compos. Anal..

[31] Liu M., Min L., Zhu C., Rao Z., Liu L., Xu W., Luo P., Fan L. (2017). Preparation, characterization and antioxidant activity of silk peptides grafted carboxymethyl chitosan. Int. J. Biol. Macromol..

[32] Chen X., Fang F., Wang S. (2020). Physicochemical properties and hepatoprotective effects of glycated Snapper fish scale peptides conjugated with xylose via maillard reaction. Food Chem. Toxicol..

[33] Limsuwanmanee J., Chaijan M., Manurakchinakorn S., Panpipat W., Klomklao S., Benjakul S. (2014). Antioxidant activity of Maillard reaction products derived from stingray (Himantura signifier) non-protein nitrogenous fraction and sugar model systems. LWT Food Sci. Technol..

[34] Chen J., Yao Y. (2023). Phytoglycogen to Enhance the Solubility and in-vitro Permeation of Resveratrol. Food Biophys..

[35] Jackson M., Mantsch H.H. (1995). The Use and Misuse of FTIR Spectroscopy in the Determination of Protein Structure. Crit. Rev. Biochem. Mol. Biol..

[36] Wang S., Zhang Y., Chen L., Xu X., Zhou G., Li Z., Feng X. (2018). Dose-dependent effects of rosmarinic acid on formation of oxidatively stressed myofibrillar protein emulsion gel at different NaCl concentrations. Food Chem..

[37] Li W., Zhao H., He Z., Zeng M., Qin F., Chen J. (2016). Modification of soy protein hydrolysates by Maillard reaction: Effects of carbohydrate chain length on structural and interfacial properties. Colloids Surf. B.

[38] Pancoska P., Wang L., Keiderling T.A. (1993). Frequency analysis of infrared absorption and vibrational circular dichroism of proteins in D2O solution. Protein Sci..

[39] Zare F., Ghaedi M., Jannesar R., Tayebi L. (2018). Switchable polarity solvents for preconcentration and simultaneous determination of amino acids in human plasma samples. New J. Chem..

[40] Wang D., Liu Y., Guo M., Sun J. (2024). Effect of Ball-Milling Treatment Combined with Glycosylation on the Structure and Functional Properties of Litopenaeus vannamei Protein. Foods.

[41] Yang Y., Cui S.W., Gong J., Guo Q., Wang Q., Hua Y. (2015). A soy protein-polysaccharides Maillard reaction product enhanced the physical stability of oil-in-water emulsions containing citral. Food Hydrocoll..

[42] He W., Tian L., Fang F., Pan S., Jones O.G. (2022). Heat-induced glycosylation with dextran to enhance solubility and interfacial properties of enzymatically hydrolyzed zein. J. Food Eng..

[43] Liu J., Ru Q., Ding Y. (2012). Glycation a promising method for food protein modification: Physicochemical properties and structure, a review. Food Res. Int..

[44] Khan H., Mudgil P., Alkaabi S.A.S., AlRashdi Y.H.S., Maqsood S. (2024). Maillard reaction-based conjugation of pea protein and prebiotic (polydextrose): Optimization, characterization, and functional properties enhancement. Front. Sustainable Food Syst..

[45] Zha F., Yang Z., Rao J., Chen B. (2019). Gum Arabic-Mediated Synthesis of Glyco-pea Protein Hydrolysate via Maillard Reaction Improves Solubility, Flavor Profile, and Functionality of Plant Protein. J. Agric. Food. Chem..

[46] Chen X., Dai Y., Huang Z., Zhao L., Du J., Li W., Yu D. (2022). Effect of ultrasound on the glycosylation reaction of pea protein isolate–arabinose: Structure and emulsifying properties. Ultrason. Sonochem..

[47] Pereira Souza A.C., Deyse Gurak P., Marczak D.F.L. (2017). Maltodextrin, pectin and soy protein isolate as carrier agents in the encapsulation of anthocyanins-rich extract from jaboticaba pomace. Food Bioprod. Process..

[48] Wang X., Liu X., Zheng X., Qu Y., Shi Y. (2020). Preparation of corn glycopeptides and evaluation of their antagonistic effects on alcohol-induced liver injury in rats. J. Funct. Foods.

[49] Lesmes U., McClements D.J. (2012). Controlling lipid digestibility: Response of lipid droplets coated by β-lactoglobulin-dextran Maillard conjugates to simulated gastrointestinal conditions. Food Hydrocoll..

[50] Petrov L., Kachaunov M., Alexandrova A., Tsvetanova E., Georgieva A., Dolashki A., Velkova L., Dolashka P. (2022). Snail Mucus Protective Effect on Ethanol-Induced Gastric Ulcers in Mice. Life.

[51] Chen B., Dong X., Zhang J., Wang W., Song Y., Sun X., Zhao K., Sun Z. (2023). Effects of oxidative stress regulation in inflammation-associated gastric cancer progression treated using traditional Chinese medicines: A review. Medicine.

[52] Labenz C., Toenges G., Wörns M.-A., Sprinzl M.F., Galle P.R., Schattenberg J.M. (2020). Liver injury in patients with severe acute respiratory syndrome coronavirus-2 infection: A systematic review and meta-analysis. Eur. J. Gastroenterol. Hepatol..

[53] Qin T., Ren Z., Liu X., Luo Y., Long Y., Peng S., Chen S., Zhang J., Ma Y., Li J. (2019). Study of the selenizing Codonopsis pilosula polysaccharides protects RAW264.7 cells from hydrogen peroxide-induced injury. Int. J. Biol. Macromol..

[54] Suzuki T., Motohashi H., Yamamoto M. (2013). Toward clinical application of the Keap1–Nrf2 pathway. Trends Pharmacol. Sci..

[55] Lu Z., Xuehong M., Rongmei S., Libo Z., Ruolin Z., Ran D., Yuanyuan Q., Sijia G., Xinxia L., Jingjing D. (2022). Allicin ameliorates IMQ-induced psoriasis-like skin inflammation via disturbing the interaction of keratinocytes with IL-17A. Br. J. Pharmacol..

[56] Hou Y., Zhang X., Liu X., Wu Q., Hou J., Su P., Guo Q. (2022). Comparison of the Effects of 5-Hydroxymethylfurfural in Milk Powder Matrix and Standard Water on Oxidative Stress System of Zebrafish. Foods.

[57] Buhrke T., Lengler I., Lampen A. (2011). Analysis of proteomic changes induced upon cellular differentiation of the human intestinal cell line Caco-2. Dev. Growth Differ..

[58] Petit E., Michelet X., Rauch C., Bertrand-Michel J., Tercé F., Legouis R., Morel F. (2009). Glutathione transferases kappa 1 and kappa 2 localize in peroxisomes and mitochondria, respectively, and are involved in lipid metabolism and respiration in Caenorhabditis elegans. FEBS J..

[59] Bonekamp N.A., Völkl A., Fahimi H.D., Schrader M. (2009). Reactive oxygen species and peroxisomes: Struggling for balance. BioFactors.

[60] Kalinina E.V., Chernov N.N., Novichkova M.D. (2014). Role of glutathione, glutathione transferase, and glutaredoxin in regulation of redox-dependent processes. Biochemistry Mosc..

[61] Sreejayan, Rao M.N.A. (2011). Curcuminoids as Potent Inhibitors of Lipid Peroxidation. J. Pharm. Pharmacol..

